# Health Outcomes and Health Services Utilization Evaluation Protocol: Assessing the Impact of the Nova Scotia Rapid Access and Stabilization Program

**DOI:** 10.1192/j.eurpsy.2024.665

**Published:** 2024-08-27

**Authors:** M. K. Adu, R. D. L. Dias, G. Obuobi-Donkor, N. Ezeanozie, S. Sridharan, J. Morrison, P. Simon, B. Taylor, M. MacKinnon, S. Gossen, B. Agyapong, L. Wozney, V. I. Agyapong

**Affiliations:** ^1^Department of Psychiatry, Dalhousie University; ^2^Mental Health and Addictions Program, Nova Scotia Health, Halifax, NS; ^3^Department of Psychiatry, University of Alberta, Edmonton, AB, Canada

## Abstract

**Introduction:**

Emergency psychiatric care, unplanned hospital admissions, and inpatient health care are the costliest forms of mental health care. According to Statistics Canada (2018), almost 18% (5.3 million) of Canadians reported needing mental health support. However, just above half of this figure (56.2%) have reported their needs were fully met. To further expand capacity and access to mental health care in the province, Nova Scotia Health has launched a novel mental health initiative, the Rapid Access, and Stabilization Program (RASP).

**Objectives:**

This study evaluates the effectiveness and impact of the RASP on high-cost health services utilization (e.g. ED visits, mobile crisis visits, and inpatient treatments) and related costs. It also assesses healthcare partners’ (e.g. healthcare providers, policymakers, community leaders) perceptions and patient experiences and satisfaction with the program and identifies sociodemographic characteristics, psychological conditions, recovery, well-being, and risk measures in the assisted population.

**Methods:**

This is a hypothesis-driven program evaluation study that employs a mixed methods approach. A within-subject comparison will examine health services utilization data from patients attending RASP, one year before and one year after their psychiatry assessment at the program. A controlled between-subject comparison will use historical data from a control population will examine whether possible changes in high-cost health services utilization are associated with the intervention (RASP). The primary analysis involves extracting secondary data from provincial information systems, electronic medical records, and regular self-reported clinical assessments. Additionally, a qualitative sub-study will examine patient experience and satisfaction, and examine health care partners’ impressions.

**Results:**

The results for the primary, secondary, and qualitative outcome measures to be available within 6 months of study completion. We expect that RASP evaluation findings will demonstrate a minimum 10% reduction in high-cost health services utilization and corresponding 10% cost savings, and also a reduction in the wait times for patient consultations with psychiatrists to less than 30 calendar days. In addition, we anticipate that patients, healthcare providers, and healthcare partners would express high levels of satisfaction with the new service.

**Conclusions:**

This study will demonstrate the results of the Mental Health and Addictions Program (MHAP) efforts to provide stepped-care, particularly community-based support, to individuals with mental illnesses. Results will provide new insights into a novel community-based approach to mental health service delivery and contribute to knowledge on how to implement mental health programs across varying contexts.

**Disclosure of Interest:**

None Declared

